# Surveillance for Lyme disease in Canada, 2009–2019

**DOI:** 10.14745/ccdr.v48i05a05

**Published:** 2022-05-05

**Authors:** Salima Gasmi, Jules K Koffi, Mark P Nelder, Curtis Russell, Scott Graham-Derham, Lisa Lachance, Bijay Adhikari, Jacqueline Badcock, Shamara Baidoobonso, Beverly A Billard, Beth Halfyard, Stéphanie Jodoin, Mayank Singal, Annie-Claude Bourgeois

**Affiliations:** 1 Centre for Food-borne, Environmental and Zoonotic Infectious Diseases, Public Health Agency of Canada, Saint-Hyacinthe, QC; 2 Enteric, Zoonotic and Vector-Borne Diseases, Public Health Ontario, Toronto, ON; 3 Communicable Disease Control Branch, Manitoba Health and Seniors Care, Winnipeg, MB; 4 Health and Wellness Promotion Branch, Public Health and Compliance Division, Alberta Health, Edmonton, AB; 5 Population Health Branch, Ministry of Health, Regina, SK; 6 Office of the Chief Medical Officer of Health, New Brunswick Department of Health, Fredericton, NB; 7 Prince Edward Island Department of Health and Wellness, Population Health Assessment & Surveillance Unit, Charlottetown, PE; 8 Public Health Branch, Nova Scotia Department of Health and Wellness, Halifax, NS; 9 Health and Community Services, Population Health Branch, St. John’s, NL; 10 Direction générale adjointe de la protection de la santé publique, Ministère de la Santé et des Services sociaux, Québec, QC; 11 Communicable Diseases and Immunization Service, BC Centre for Disease Control, Vancouver, BC; 12 Centre for Food-borne, Environmental and Zoonotic Infectious Diseases, Public Health Agency of Canada, Ottawa, ON

**Keywords:** surveillance, *Ixodes scapularis*, *Ixodes pacificus*, tick-borne disease, Lyme disease, Canada

## Abstract

**Background:**

Lyme disease (LD) is a multisystem infection that can affect the skin, heart, joints and nervous system. In Canada, the incidence of LD cases has increased over the past decade making this a disease of public health concern. The objective of this study is to summarize the epidemiology of LD cases reported in Canada from 2009 through 2019.

**Methods:**

Incidence over time, case classification (confirmed and probable), seasonal and geographic distribution, demographic and clinical characteristics of reported LD cases were determined. Logistic regression was used to explore potential demographic risk factors for the occurrence of LD.

**Results:**

During 2009–2019, a total of 10,150 LD cases were reported by the provinces to the Public Health Agency of Canada, of which 7,242 (71.3%) were confirmed and 2,908 (28.7%) were probable cases. The annual count increased from 144 in 2009 to 2,634 in 2019, mainly due to an increase in locally acquired infections, from 65.3% to 93.6%, respectively. The majority of cases (92.1%) were reported from three provinces: Ontario (46.0%); Nova Scotia (28.0%); and Québec (18.1%). Most of the locally acquired cases (74.0%) were reported in the summer months of June (20.0%), July (35.4%) and August (18.6%). The highest incidence rates (cases per 100,000 population) were in children aged 5–9 years (45.0) and in adults aged 65–69 years (74.3), with 57.3% of all reported cases occurring among males. The most common presenting symptoms were single erythema migrans rash (75.1%) and arthritis (34.1%). The frequency of reported clinical manifestations varied among age groups and seasons with erythema migrans and arthritis at presentation reported more frequently in children than older patients.

**Conclusion:**

The results of this report highlight the continued emergence of LD in Canada and the need for further development and implementation of targeted awareness campaigns designed to minimize the burden of LD.

## Introduction

Lyme disease (LD) is the most commonly reported tick-borne zoonosis in North America and Europe. In Canada, LD is caused by the spirochete *Borrelia burgdorferi* (*B. burgdorferi*) *sensu stricto* and transmitted by *Ixodes pacificus* (*I. pacificus*) ticks in British Columbia and *Ixodes scapularis* (*I. scapularis*) in central and eastern Canada. Over the last decade, the warming climate as well as anthropogenic factors such as landscape changes, have contributed to tick and tick-borne diseases expanding their geographic range ( ((1,2))). As a result, the incidence of LD cases increased over the past decade ( ((3))) making this a disease of public health concern in Canada.

Lyme disease is a multisystem infection that can affect the skin, heart, joints and nervous system. Approximately 70% of people bitten by a *B. burgdorferi*-infected tick will develop a cutaneous rash, erythema migrans, which may be accompanied by flu-like symptoms ( ((4))). If left untreated, spirochetes disseminate throughout the body via the blood and may cause multiple secondary erythema migrans lesions, cardiac manifestations (carditis, atrioventricular heart block, arrhythmia and palpitations) and neurologic manifestations (facial paralysis—Bell’s palsy—and meningitis). Months to years post-infection, late LD can manifest with single or recurrent joint arthritis episode(s). It is noteworthy that one death attributed to complications of Lyme carditis has been recorded in Canada in 2018 ( ((5))).

This report summarizes the epidemiology of LD cases reported in Canada from 2009 through 2019.

## Methods

### Case definition

Lyme disease became nationally notifiable in 2009. In 2016, the LD case definition ( ((6))) was revised to propose five methods to identify LD risk to simplify reporting by jurisdictions (**Table A1**).

### Data sources

Information on reported LD cases from 2009 through 2019 was obtained from the provincial and territorial public health authorities via the Canadian Notifiable Disease Surveillance System (CNDSS) and the Lyme Disease Enhanced Surveillance (LDES) system of the Public Health Agency of Canada (PHAC). The CNDSS only collects demographic data, episode date and case classification from the provinces and territories. The LDES system captures additional data, including possible geographic location of exposure for both locally acquired and travel-related cases, clinical manifestations and results of laboratory testing. Public health units in the provinces and territories are responsible for investigating the cases reported by clinicians using a case management tool ( ((7))). They collect among other information, the most likely location of LD acquisition, whether in Canada or abroad, regardless of the stage of disease ( ((8))).

### Analysis

Incidence rates per 100,000 population for reported cases were calculated by year, province, age group and sex using the census population estimates for July 1^st^ of Statistics Canada data ( ((9))) for each year of the reporting period, 2009–2019. Seasonality was determined by the reported date of the symptom onset. Percentages of reported clinical manifestations of locally acquired infections were calculated for overall cases and by age group. The most likely geographic location for acquisition of LD infection was superimposed on a map of LD risk areas ( ((6))). Cases with a history of travel (within or outside of Canada) within 30 days of reporting were not included in the analysis of geographic distribution.

For locally acquired cases with no missing data, variations among age groups, sex, month of onset and reporting year were explored in multivariate logistic regression using Stata, version 15.1 (StataCorp, College Station, Texas, United States). In separate models, the binary outcome variable was the presence or absence of LD stage at presentation, for each of early localized (single erythema migrans), early disseminated (multiple erythema migrans, cardiac manifestations, Bell’s palsy and other neurological manifestations) and late disseminated stage (arthritis) as classified by the Infectious Disease Society of America guidelines ( ((10))). For each model, explanatory variables were age group (10 and 15-year intervals), sex, month of symptom onset (in four categories for simplicity), year and province of reporting. The explanatory variable “province” was included in the analysis to account for possible variability in reporting between provinces. Explanatory variables were screened in bivariable logistic regression models, and those significant at the level of *p*<0.1 were included in multivariable models. The most parsimonious multivariate models were sought by backward elimination of non-significant variables until all factors in the model were significant (*p*<0.05).

## Results

### Incidence over time

**Figure 1:**
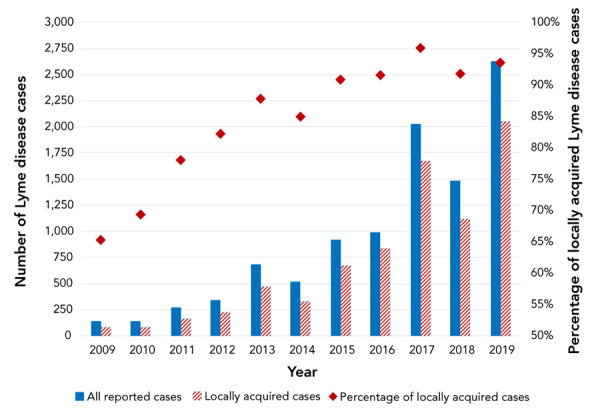
Number and proportion of Lyme disease cases, all reported and locally acquired (percentage^a^) in Canada, 2009−2019 a Percentage of locally acquired cases among those cases with known location of probable acquisition in Canada

From 2009 through 2019, 10,150 LD cases were reported in Canada. Of these 7,242 (71.3%) were confirmed and 2,908 (28.7%) were probable cases (**Table 1**). Overall, the annual number of reported cases increased from 144 in 2009 to 2,634 in 2019 (incidence rates per 100,000 population of 0.4 and 7.0, respectively); however, in 2014 and 2018 the number of cases decreased (**Table A2**). The number of cases acquired in Canada increased from 79 to 2,052 during the same period, representing 65.3% and 93.6%, respectively, of cases with known exposure location (**Figure 1**).

**Table 1:** Classification (confirmed and probable) of reported Lyme disease casesa, 2009–2019

Classification	Reported Lyme disease cases																							
	Year																							
	2009		2010		2011		2012		2013		2014		2015		2016		2017		2018		2019		Total	
	N	%	N	%	N	%	N	%	N	%	N	%	N	%	N	%	N	%	N	%	N	%	N	%
All cases (n=10,150)																								
Confirmed	115	79.9	109	76.2	109	76.2	232	68.6	485	71.1	334	64.0	651	71.0	672	67.7	1,496	73.9	1,053	70.8	1,900	72.2	7,242	71.3
Probable	29	20.1	34	23.8	34	23.8	106	31.4	197	28.9	188	36.0	266	29.0	320	32.3	529	26.1	434	29.2	734	27.8	2,908	28.7
Total	144	100	143	100	143	100	338	100	682	100	522	100	917	100	992	100	2,025	100	1,487	100	2,634	100	10,150	100
Cases acquired in Canada (n=7,691)																								
Confirmed	56	70.9	56	65.1	56	65.1	129	58.1	286	61.1	198	59.5	467	70.0	542	64.8	1,204	72.0	751	67.2	1,410	68.7	5,195	67.5
Probable	23	29.1	30	34.9	30	34.9	93	41.9	182	38.9	135	40.5	200	30.0	294	35.2	467	28.0	366	32.8	642	31.3	2,496	32.5
Total	79	100	86	100	86	100	222	100	468	100	333	100	667	100	836	100	1,671	100	1,117	100	2,052	100	7,691	100

**Table:** 

a Acquired in Canada: cases with no history of travel outside of Canada, within 30 days of reporting

Among all reported cases, the majority (92.1%) were reported from three provinces: Ontario (46.0%); Nova Scotia (28.0%); and Québec (18.1%). In 2019, Nova Scotia reported a LD incidence that was twelve-fold greater than the LD incidence for Canada overall (85.6 versus 7.0 per 100,000 population) (**Table 2**).

**Table 2:** Incidence per 100,000 population of Lyme disease cases reported by province and year in Canada^a^, 2009–2019

Province	Incidence per 100,000 population										
	2009	2010	2011	2012	2013	2014	2015	2016	2017	2018	2019
All cases (n=10,150)											
British Columbia	0.2	0.2	0.4	0.4	0.1	0.1	0.4	0.8	0.3	0.2	0.3
Alberta	0.0	0.0	0.2	0.2	0.5	0.2	0.3	0.2	0.3	0.3	0.3
Saskatchewan	0.0	0.0	0.1	0.0	0.1	0.0	0.0	0.1	0.3	0.2	0.1
Manitoba	0.4	1.0	1.0	1.5	2.3	2.7	2.4	3.9	3.2	4.0	4.7
Ontario	0.8	0.7	1.0	1.4	2.4	1.7	3.1	2.7	7.1	4.4	8.0
Québec	0.2	0.1	0.4	0.5	1.7	1.5	1.9	2.2	4.0	3.6	5.9
New Brunswick	0.0	0.3	0.7	0.9	0.7	0.7	1.7	1.4	3.9	2.6	4.6
Nova Scotia	1.7	1.8	5.7	5.4	16.2	12.1	26.1	34.6	61.2	47.0	85.6
Prince Edward Island	0.0	0.0	0.7	1.4	0.0	0.0	2.7	2.7	2.0	0.7	3.8
Newfoundland & Labrador	0.0	0.2	0.0	0.0	0.0	0.0	0.4	0.2	0.0	0.4	0.0
Canada	0.4	0.4	0.8	1.0	1.9	1.5	2.6	2.7	5.5	4.0	7.0
Cases acquired in the province of residency (n=7,200)											
British Columbia	N/A	N/A	N/A	N/A	N/A	N/A	N/A	0.1	0.1	0.0	0.0
Saskatchewan	N/A	N/A	N/A	N/A	N/A	N/A	N/A	0.0	0.1	0.0	0.0
Manitoba	0.3	0.6	0.6	1.0	2.0	2.4	2.3	2.4	2.9	3.5	4.1
Ontario	0.5	0.5	0.8	0.8	2.1	1.3	2.7	2.0	5.5	2.5	5.0
Québec	N/A	N/A	N/A	N/A	N/A	N/A	N/A	1.4	3.0	2.7	3.8
New Brunswick	0.0	0.3	0.4	0.7	0.7	0.5	1.5	0.8	3.5	2.5	3.1
Nova Scotia	1.5	1.5	5.2	5.3	16.1	12.1	26.1	25.4	55.2	34.8	79.2
Prince Edward Island	0.0	0.0	0.0	0.7	0.0	0.0	0.0	0.0	0.0	0.0	0.0

Abbreviation: N/A, data on probable location of Lyme disease acquisition not available

^a^ No case has been reported from Yukon, Northwest Territories and Nunavut. All Lyme disease cases reported from Alberta and Newfoundland and Labrador were travel-related only. Data on location of acquisition were available for 83.2% (n=8,444) of all reported cases

During 2009–2019, data on history of travel was available for 83.2% (n=8,444) of all reported cases (n=10,150). Of the cases acquired during travel outside Canada, 363 (57.4%) were exposed in the United States and 261 (41.3%) in Europe.

### Seasonal distribution

**Figure 2:**
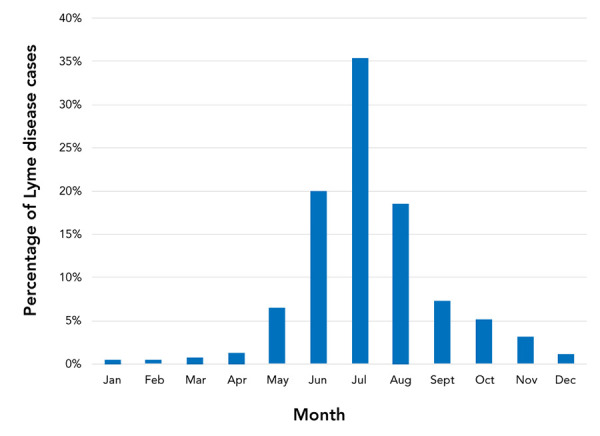
Month of illness onseta of Lyme disease cases acquired in Canada, 2009−2019 a Month of illness onset is the month of first symptoms seen

Over the study period, the month of illness onset for locally acquired cases was available for 6,278 cases (81.6%). Most cases (95.8%) were reported from May through November, with the majority reported in the summer months of June (20.0%), July (35.4%) and August (18.6%) (**Figure 2**).

### Geographic distribution

Information on location of LD acquisition (at the sub-provincial level) was available for 93.5% (n=6,734) of the locally acquired cases. Most of the cases were concentrated in locations in southern Manitoba, south-central and south-eastern Ontario, southern Québec and New Brunswick and in Nova Scotia.

**Figure 3:**
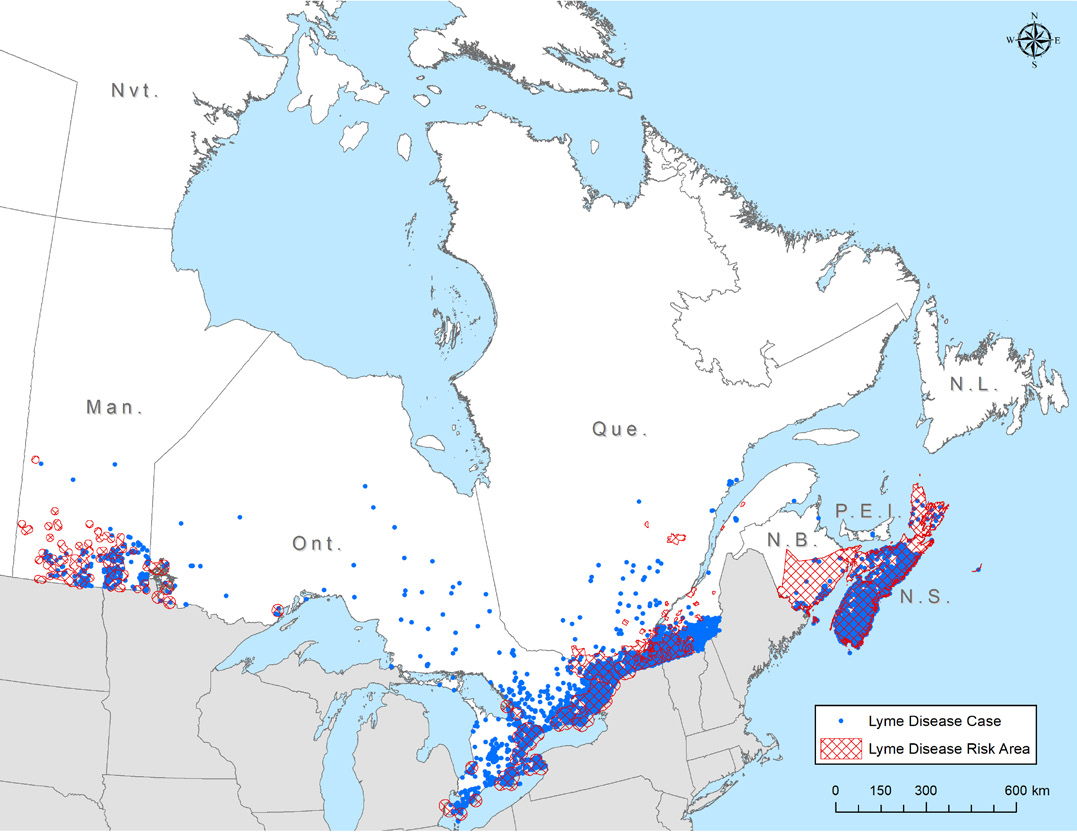
Reported locations of Lyme disease acquisitiona, Canada, 2009–2019 ^a^ Each dot on the map represents the probable location of infection acquisition, randomly distributed at the census subdivision level for all provinces except: Québec (2016–2018) and Nova Scotia (2019) used the administrative region and forward sortation area (FSA) of residency, respectively. In 2018, all of Nova Scotia was declared at risk of Lyme disease, and since then, the probable location of acquisition was based on the FSA of residency for cases with no travel history outside the province. Data on location of acquisition was not available at sub-provincial level for British Columbia and Saskatchewan; Saskatchewan reported one locally acquired case in 2017. Cases reported by Alberta and Newfoundland & Labrador were travel-related only. Hatched areas indicate Lyme disease risk areas. These are locations where surveillance activities suggest that populations of the Lyme disease vector, *Ixodes scapularis* have been established and the likely transmission of *B. burgdorferi* is occurring

Some cases were likely acquired outside locations where populations of *I. scapularis* ticks are established (the hatched areas in the **Figure 3**).

### Demographic and clinical characteristics

Demographic information was available for 7,667 (99.7%) of reported locally acquired LD cases. The overall average age was 47.4 years (95% confidence interval (CI): 46.9–47.9).

**Figure 4:**
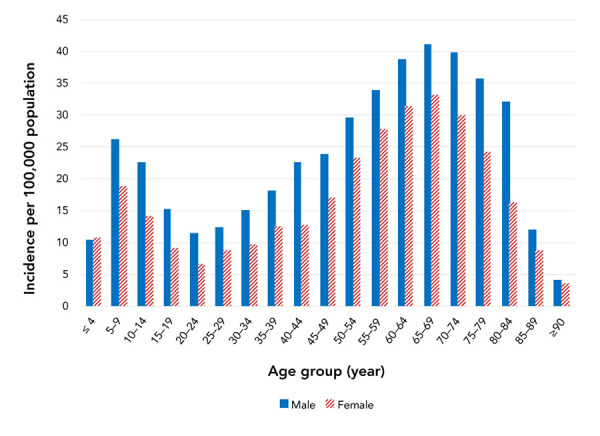
Cumulative incidencea per 100,000 population of Lyme disease cases by age group and sex, Canada, 2009–2019 (n=7,667) a The denominators used to calculate the incidences were obtained from Statistics Canada, population estimates on July 1^st^

The cumulative incidence per 100,000 population shows a bimodal pattern with peaks in children aged 5–9 years (45.0) and adults aged 65–69 years (74.3). Incidence was higher in adults aged 50−84 years, representing 57.0% of all reported cases (**Figure 4**).

Lyme disease cases were reported more often in males than females (57.3% versus 42.7%). In all age groups, incidence was higher among males than females, except for children under five years where the incidence was similar (Figure 4).

Information on clinical manifestations of LD cases was available for 4,961 (64.5%) of locally acquired infections. The most commonly reported manifestations were single erythema migrans rash (75.1%) and arthritis (34.1%). Cardiac manifestations, Bell’s palsy and other neurologic manifestations were reported in 3.7%, 7.6% and 19.0% of cases, respectively. Among the 4,961 cases, 33.7% reported a combination of LD stages; 28.2% had early localized stage and early disseminated or late disseminated stage, and 5.5% had early and late disseminated stages.

In a multivariate analysis of the LD stages at presentation (**Table 3**), the odds of age groups 10−19, 20−29, 30−39 and 50−59 years for being diagnosed in early localized stage were respectively 0.56 (95% CI: 0.42−0.75), 0.63 (95% CI: 0.47−0.85), 0.60 (95% CI: 0.46−0.79) and 0.77 (95% CI: 0.61−0.97) times lower than the reference age group 0−9 years.

**Table 3:** Final multivariate binomial logistic regression models testing the factors that influence the occurrence of Lyme disease stagesa^,b^ of locally acquired cases, Canada, 2009–2019 (n=4,913)

Outcome	Explanatory variables	Odds ratio		Wald (*z* value)	*p*-value
		n	95% CI		
Early localized	10–19 years vs 0–9 years	0.56	0.42–0.75	-3.96	0.00
	20–29 years vs 0–9 years	0.63	0.47–0.85	-3.05	0.00
	30–39 years vs 0–9 years	0.60	0.46–0.79	-3.65	0.00
	50–59 years vs 0–9 years	0.77	0.61–0.97	-2.21	0.03
	Dec–Feb vs Jun–Aug	0.46	0.30–0.69	-3.77	0.00
Early disseminated	10–19 years vs 0–9 years	1.56	1.09–2.25	2.41	0.02
	20–29 years vs 0–9 years	1.76	1.21–2.55	2.98	0.00
	60–69 years vs 0–9 years	1.42	1.04–1.94	2.19	0.03
	Sep–Nov vs Jun–Aug	0.81	0.66–0.99	-2.08	0.04
	Dec–Feb vs Jun–Aug	0.51	0.29–0.90	-2.32	0.02
	Mar–May vs Jun–Aug	0.62	0.46–0.82	-3.32	0.00
Late disseminated	60–74 years vs 0–14 years	0.78	0.64–0.96	-2.36	0.02
	75 or more years vs 0–14 years	0.52	0.38–0.72	-4.04	0.00
	Male vs female	1.14	1.01–1.29	2.10	0.04
	Sep–Nov vs Jun–Aug	1.29	1.09–1.53	3.00	0.00
	Dec–Feb vs Jun–Aug	3.30	2.23–4.88	5.99	0.00
	Mar–May vs Jun–Aug	1.35	1.09–1.67	2.73	0.01

Abbreviations: CI, confidence intervals; vs, versus

a Clinical manifestations of Lyme disease classified as defined by the Infectious Disease Society of America guidelines ( ((8)))

^b^ Early localized (single erythema migrans), early disseminated (multiple erythema migrans, cardiac manifestations, Bell’s palsy and other neurological manifestations) and late disseminated (arthritis)

When the outcome was in the early disseminated stage, the odds of age groups 10−19, 20−29 and 60−69 years were, respectively, 1.56 (95% CI: 1.09−2.25), 1.76 (95% CI: 1.21−2.55) and 1.42 (95% CI: 1.04−1.94) times higher compared with the 0–9 years age group. In contrast, when the outcome was in late disseminated stage, the odds of age groups 60−74 and 75 years and older were respectively 0.78 (95% CI: 0.64−0.96) and 0.52 (95% CI: 0.38−0.78) times lower when compared to the reference age group, 0–14 years.

The number of cases with early localized stage was less likely to be reported from December–February versus June–August (odds ratio [OR]: 0.46; 95% CI: 0.30−0.69). Cases with early disseminated stage were less likely to be reported September−November (OR: 0.81; 95% CI: 0.66−0.99), December−February (OR: 0.51; 95% CI: 0.29−0.90) and March−May (OR: 0.62; 95% CI: 0.46−0.82) than in the summer months of June–August. Cases diagnosed in late disseminated stage were reported more often during September–November (OR: 1.29; 95% CI: 1.09−1.53), December−February (OR: 3.30; 95% CI: 2.23−4.88) and March−May (OR: 1.35; 95% CI: 1.09−1.67) when compared with June–August.

Males were more likely to be reported with late disseminated stage than females (OR: 1.14; 95% CI: 1.01−1.29).

## Discussion

This report provides an update on the epidemiology of LD cases reported in Canada from 2009 through 2019. Over the 11-year period, the incidence of reported LD cases has increased dramatically in Canada, mainly due to an increase in the number of locally acquired infections. The vast majority of cases continued to be concentrated in southern Manitoba, south-central and south-eastern Ontario, southern Québec and New Brunswick and in Nova Scotia, which likely coincide in most cases with the areas where *I. scapularis* ticks carrying *B. burgdorferi* are established ( ((11))).

In these parts of the country, the tick vector is spreading northward as a result of climate warming that drives, in part, the growing suitable habitat for tick survival and establishment ( ((12))). Moreover, greater awareness among the public and healthcare providers and improvements to public health surveillance and notification may also have contributed to the increase in the number of reported cases.

It is important to note that the incidence in Nova Scotia during 2019 was twelve-fold higher than the national incidence. This is most likely a result of the increased number of established blacklegged tick populations; including the density, geographic range, *B. burgdorferi* infection prevalence ( ((13))), and the maritime climate that permits, during some warm spells in winter, tick activity in the absence of snow cover ( ((14,15))).

In contrast, in western Canada, where the predominant vector *I. pacificus* is distributed along the coast and southern region of British Columbia, the LD risk has remained relatively low and stable because of the low prevalence of infected ticks ( ((16))).

Lyme disease symptom onset was more likely reported from May to November, a pattern that corresponds to nymphal tick activity in the summer months ( ((17))); these time periods also overlap with when people are most engaged in outdoor activities that expose them to the risk of tick bites and *B. burgdorferi* transmission. However, some cases reported illness onset in winter months, which may be explained by 1) the occurrence of the late disseminated stage that appears months to years post-infection in untreated patients ( ((18))) or 2) advantageous weather that allows tick activity in British Columbia ( ((19))).

Consistent with previous studies, the age distribution of cases was bimodal in children and older adults. Among children, incidence peaked in those aged 5–9 years, which corroborates previous studies in Canada and the United States ( ((20–22))). However, we found that adults aged 50−84 years were at higher risk of LD compared with previous studies, which reported younger groups (range 50−64 years) to be the most at risk ( ((20–23))). The level of adoption of preventive behaviours toward LD or to the level of tick exposure could have contributed to the observed discrepancy in the most at-risk age group for LD ( ((24))). Targeted education and awareness of preventive measures are needed for older persons in order to decrease the risk of tick bites and LD.

Erythema migrans and arthritis were the most commonly reported clinical manifestations in this study; consistent with findings reported previously in Canada and the Unite States ( ((20–23))). Lyme arthritis is a manifestation of late-stage LD that usually appears in 51% of untreated patients ( ((25))), highlighting the importance of early detection and treatment.

Males were more likely to have LD than females across almost all age groups, similar to previous reports from Canada and the United States ( ((20–22))). Additionally, late disseminated stage was more common in males than females, although no significant differences were found for earlier disease stages. This apparent sex-based difference might be a result of higher risk of tick bites ( ((26))) and LD transmission; however, the reason why males are diagnosed more often than females with late Lyme arthritis may be due to a difference in immunologic response to *B. burgdorferi* infection ( ((27))) or simply to a delay in presenting for medical care. Further research is needed to elucidate this finding.

**Figure A1:**
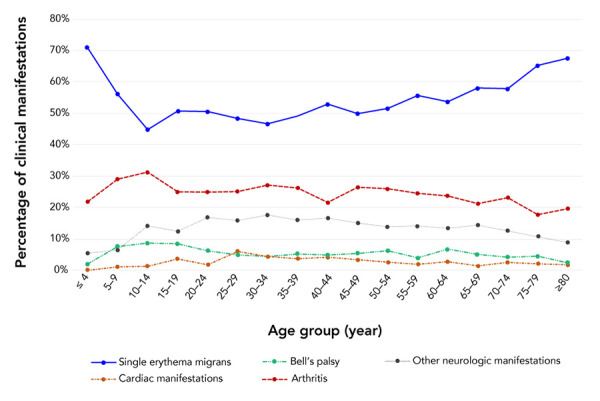
Percentage of clinical manifestations by age group for locally acquired Lyme disease cases, in Canada, 2009−2019 (n=4,951)

Across age categories, the percentage of reported clinical manifestations varied widely in children younger than 15 years. The percentage of cases with single erythema migrans (early localized stage) was higher in younger children and the proportion of arthritis (late disseminated stage) was higher in older children (**Figure A1**) which is consistent with a study from the Canadian Paediatric Surveillance Program ( ((14))). Furthermore, children are at higher risk of both early localized LD and Lyme arthritis than older patients. Studies have found that children are at higher risk of tick bites from vector ticks such as *I. scapularis* and *I. pacificus* ( ((26,28,29))); hence, efforts to enhance awareness of ticks and LD should target this at-risk age group and their parents and caregivers.

The LD stage at presentation varied significantly between seasons. Cases with early localized stage were more frequently reported in summer months (June–August) than winter months (December–February); and cases with arthritis were less frequently reported in summer months compared with the rest of the year. This is expected given that untreated patients have manifestations of the early localized stage that appear within 30 days of infection, and the late disseminated stage can appear months to years post infection ( ((24))). In contrast, cases with early disseminated stage were diagnosed more in the summer than other parts of the year. Given that early disseminated stage appears in untreated patients within three months post infection, this finding suggests it is likely that there is some underreporting of cases with cardiac and neurologic manifestations, which may go undetected or unreported outside of the summer months. This result underscores that prevention messages should not only be focused during the spring and summer months when nymphal and adult ticks are most active, but year-round.

### Limitations

There are several limitations to the interpretation of the findings of this report. First, it is likely that the incidence rates over time are conservative estimates as some LD cases may be undiagnosed and probable cases may be underreported. Second, the 2016 LD case definition revision could have impacted the reporting of some cases. Third, clinical manifestations are reported voluntarily to the provincial public health organizations, which could have led to misclassification and underreporting. Fourth, information on whether the LD infection was locally acquired or travel-related is only an estimate because not all provinces provided these data. Finally, because of limited resources, field tick surveillance to detect the expansion of LD risk areas may not be up to date in many locations, which would affect the classification of cases.

## Conclusion

The number of reported LD cases has continued to increase in Canada over the last decade, as did the geographic range of ticks that carry the LD bacterium. Continued surveillance, preventive strategies as well as early disease recognition and treatment will continue to minimize the impact of LD in Canada.

The key findings of this report highlight the need for further targeted awareness campaigns designed to minimize the burden of LD in Canada.

## Annex

**Table A1:** 2016 Lyme disease case definition

Confirmed case	Probable case
Clinical evidence of illness with laboratory confirmation by one of the following methods: Isolation of *Borrelia burgdorferi* (*B. burgdorferi*) from a clinical specimen as specified by current guidelines ( ((10,18,30))) Detection of *B. burgdorferi* DNA by PCR testing on synovial fluid, cerebrospinal fluid, EM tissue biopsies or blood and by methods specified by current guidelines ( ((10,18,30))) OR Clinical evidence of illness with a history of residence in, or visit to, a Lyme disease risk area; and with laboratory evidence of infection in the form of a positive serologic test using the two-tiered approach. The two-tiered testing approach consists of a screening ELISA followed by an immunoblot assay. Immunoblots include traditional Western blots ( ((30))) or newer line blots, and both formats target an identical set of *B. burgdorferi* immunoreactive proteins ( ((31)))^a^	Clinical evidence of illness without a history of residence in, or visit to, a Lyme disease risk area; and with laboratory evidence of infection in the form of a positive serologic test as defined above under confirmed cases^a^ OR Clinician-observed erythema migrans without laboratory evidence but with history of residence in, or visit to, a Lyme disease risk area

Abbreviations: DNA, deoxyribonucleic acid; ELISA, enzyme-linked immunosorbent assay; EM, erythema migrans; PCR, polymerase chain reaction

a Laboratory comments: Criteria for serologic testing are described by the guidelines of the Canadian Public Health Laboratory Network ( ((26))). Serologic evidence is confirmatory only in patients with objective clinical evidence of disseminated Lyme disease, and a history of residence in, or visit to, a Lyme disease risk area. Serologic testing is not recommended in patients with early localized Lyme disease with exposure from a Lyme disease risk area

**Table A2:** Number of Lyme disease cases reported in Canada^a^ by province and year, 2009−2019

Province	Lyme disease cases (n=10,150)											
	2009	2010	2011	2012	2013	2014	2015	2016	2017	2018	2019	Total
British Columbia	9	7	20	18	6	5	21	40	17	9	14	166
Alberta	0	0	7	8	19	9	14	10	12	15	14	108
Saskatchewan	0	0	1	0	1	0	0	1	4	2	1	10
Manitoba	5	12	12	19	29	35	31	51	43	54	65	356
Ontario	100	93	134	191	327	229	426	371	1,005	628	1,168	4,672
Québec	14	11	32	42	142	125	160	177	329	305	500	1,837
New Brunswick	0	2	5	7	5	5	13	11	30	20	36	134
Nova Scotia	16	17	54	51	153	114	246	326	582	451	830	2,840
Prince Edward Island	0	0	1	2	0	0	4	4	3	1	6	21
Newfoundland & Labrador	0	1	0	0	0	0	2	1	0	2	0	6
Canada	144	143	266	338	682	522	917	992	2,025	1,487	2,634	10,150

^a^ No case was reported from Yukon, Northwest Territories or Nunavut to the Canadian Notifiable Disease Surveillance System of the Public Health Agency of Canada, 2009−2019
